# Adult neurogenic deficits in HIV-1 Tg26 transgenic mice

**DOI:** 10.1186/s12974-018-1322-2

**Published:** 2018-10-12

**Authors:** Raj Putatunda, Yonggang Zhang, Fang Li, Xiao-Feng Yang, Mary F Barbe, Wenhui Hu

**Affiliations:** 1 Center for Metabolic Disease Research, Temple University Lewis Katz School of Medicine, 3500 N Broad Street, Philadelphia, PA 19140 USA; 2 Department of Pathology and Laboratory Medicine, Temple University Lewis Katz School of Medicine, 3500 N Broad Street, Philadelphia, PA 19140 USA; 3 Department of Anatomy and Cell Biology, Temple University Lewis Katz School of Medicine, 3500 N Broad Street, Philadelphia, PA 19140 USA; 4 Department of Pharmacology, Temple University Lewis Katz School of Medicine, 3500 N Broad Street, Philadelphia, PA 19140 USA

**Keywords:** HIV-1, HAND, Tg26 mouse, Neurogenesis, Neural stem cells, Stemness, Differentiation, Dendritic spine density

## Abstract

**Background:**

Even in the antiretroviral treatment (ART) era, HIV-1-infected patients suffer from milder forms of HIV-1-associated neurocognitive disorders (HAND). While the viral proteins Tat and gp120 have been shown to individually inhibit the proliferation and neural differentiation of neural stem cells (NSCs), no studies have characterized the effects of all the combined viral proteins on adult neurogenesis.

**Methods:**

The HIV-1 Tg26 transgenic mouse model was used due to its clinical relevance to ART-controlled HIV-1-infected patients who lack active viral replication but suffer from continuous stress from the viral proteins. Quantitative RT-PCR analysis was performed to validate the expression of viral genes in the neurogenic zones. In vitro stemness and lineage differentiation assays were performed in cultured NSCs from HIV-1 Tg26 transgenic mice and their wild-type littermates. Hippocampal neurogenic lineage analysis was performed to determine potential changes in initial and late differentiation of NSCs in the subgranular zone (SGZ). Finally, fluorescent retroviral labeling of mature dentate granule neurons was performed to assess dendritic complexity and dendritic spine densities.

**Results:**

Varying copy numbers of partial *gag* (p17), *tat* (unspliced and spliced variants), *env* (gp120), *vpu*, and *nef* transcripts were detected in the neurogenic zones of Tg26 mice. Significantly fewer primary neurospheres and a higher percentage of larger sized primary neurospheres were generated from Tg26 NSCs than from littermated wild-type mouse NSCs, implying that Tg26 mouse NSCs exhibit deficits in initial differentiation. In vitro differentiation assays revealed that Tg26 mouse NSCs have reduced neuronal differentiation and increased astrocytic differentiation. In the SGZs of Tg26 mice, significantly higher amounts of quiescent NSCs, as well as significantly lower levels of active NSCs, proliferating neural progenitor cells, and neuroblasts, were observed. Finally, newborn mature granule neurons in the dentate gyri of Tg26 mice had deficiencies in dendritic arborization, dendritic length, and dendritic spine density.

**Conclusions:**

Both in vitro and in vivo studies demonstrate that HIV-1 Tg26 mice have early- and late-stage neurogenesis deficits, which could possibly contribute to the progression of HAND. Future therapies should be targeting this process to ameliorate, if not eliminate HAND-like symptoms in HIV-1-infected patients.

## Background

Since the start of the antiretroviral treatment (ART) era, HIV-1-associated co-morbidities have manifested in the clinical population, most notably HIV-associated neurocognitive disorders (HAND), accelerated aging, cardiovascular diseases, and metabolic dysfunction [1]. HAND continues to affect over 50% of all HIV-1-infected patients, even while undergoing ART treatment [2, 3]. This disorder describes a specific spectrum of neurocognitive impairments such as asymptomatic neurocognitive impairment (ANI), mild neurocognitive disorder (MND), and HIV-associated dementia (HAD) [4]. Although the incidence of HAD has decreased due to ART, the incidence of MND continues to rise [5–8]. It is widely accepted that a key contributing factor of neuronal dysfunction in HIV-1 infection is attributed to the “Trojan Horse” mechanism of HIV-1 neuroinvasion via infected immune cells into the central nervous system (CNS) [9–11]. However, recent reports point to the possibility that chronic neuroinflammation from HIV-1 negatively impacts adult neurogenesis, thus contributing to the evolution of HAND [2, 12].

Neurogenesis describes the process in which neuronal and glial cells are generated from neural precursors that includes neural stem cells (NSCs) and neural progenitor cells (NPCs). This process takes place during prenatal development and throughout adult life [13, 14]. In the context of adult neurogenesis, there are two main neurogenic niches: the subgranular zone (SGZ) in the dentate gyrus of the hippocampus and the subventricular zone (SVZ) lining the lateral ventricles [15–17]. In both neurogenic niches, slowly proliferating NSCs differentiate into rapidly proliferating NPCs, which then differentiate into neuroblasts and glioblasts that form neurons and glial cells respectively. Subsequently, these newborn neurons integrate into neural circuits to modulate olfactory processing and memory acquisition/maintenance [16].

Neurogenesis is important to study in the context of HAND, because HIV-1 virions have been found in the hippocampal formation of pediatric AIDS patients [18], and impaired neurogenesis has been observed in both HIV-1-infected patients and SIV-infected macaques [19, 20], as well as glial fibrillary acidic protein (GFAP)-driven gp120 transgenic mice [21–25] and GFAP-Tat transgenic mice [26, 27]. More importantly, NSCs have been shown to be targets of active HIV-1 infection [25, 27–32]. Additionally, well-known antiretroviral drugs such as AZT, efavirenz, and a tenofovir/emtricitabine/raltegravir cocktail inhibit NSC proliferation and differentiation in vitro and in vivo at pharmacologically relevant doses [33–35].

While previous studies hallmark the roles of active viral infection or viral protein production in neurogenic dysfunction [22, 24, 25, 27, 31], the role of chronic/latent HIV-1 infection in the CNS remains poorly understood. Here, we utilized the HIV-1 Tg26 mouse model to evaluate adult neurogenesis. The Tg26 mouse line expresses seven of the nine HIV-1 viral proteins under the viral long terminal repeat (LTR) promoter [36–39]. Because the replication-deficient proviral HIV-1 DNA randomly integrates into the host genome and the viral transcripts are spontaneously driven by the LTRs, the Tg26 mice serve as an appropriate model for studying the long-term effects of viral proteins on the host. This model is clinically relevant to ART-controlled HIV-1-infected patients who lack active viral replication, but suffer from continuous stress from HIV-1 viral protein exposure. The aim of this study is to characterize the effects of the combined HIV-1 viral proteins on adult neurogenesis.

## Methods

### Transgenic mice

The Institutional Animal Care and Use Committee (IACUC) at Temple University (Philadelphia, PA) approved all procedures detailed in this study that required the use of vertebrate animals prior to initiating any experimental objectives. Additionally, all methods were performed in full compliance with Temple University’s IACUC policies and the National Institutes of Health (NIH) ethical guidelines. Inbred HIV-1 Tg26 transgenic mice (Jackson Lab, #022354) and their wild-type (WT) gender and age-matched littermates were utilized in this study. These mice harbor truncated HIV-1 NL4-3 genome with a 3.1-kb deletion in the *Gag* and *Pol* regions, rendering the latent provirus replication deficient [36]. The Tg26 mice were originally generated on the FV/B background and develop a well-characterized kidney disease. As a result, most mice are moribund between 2 and 6 months of age [36, 37]. Since Tg26 mice on the C57BL/6J background do not develop kidney disease and have longer life expectancies [40–42], we generated Tg26 mice on a complete C57BL/6J background by backcrossing FVB/N-Tg(HIV)26Aln/PkltJ mice [36] with C57BL/6J mice (Jackson Lab, #000664) for at least eight generations. Since Tg26 homozygous (+/+) mice are runted and rarely survive to weaning [36], the Tg26 mice were maintained as heterozygotes (+/−) throughout the study. Mice were utilized between 8 and 12 weeks old for all the proposed studies.

### Quantitative reverse transcription PCR (RT-qPCR)

**Fig. 1:**
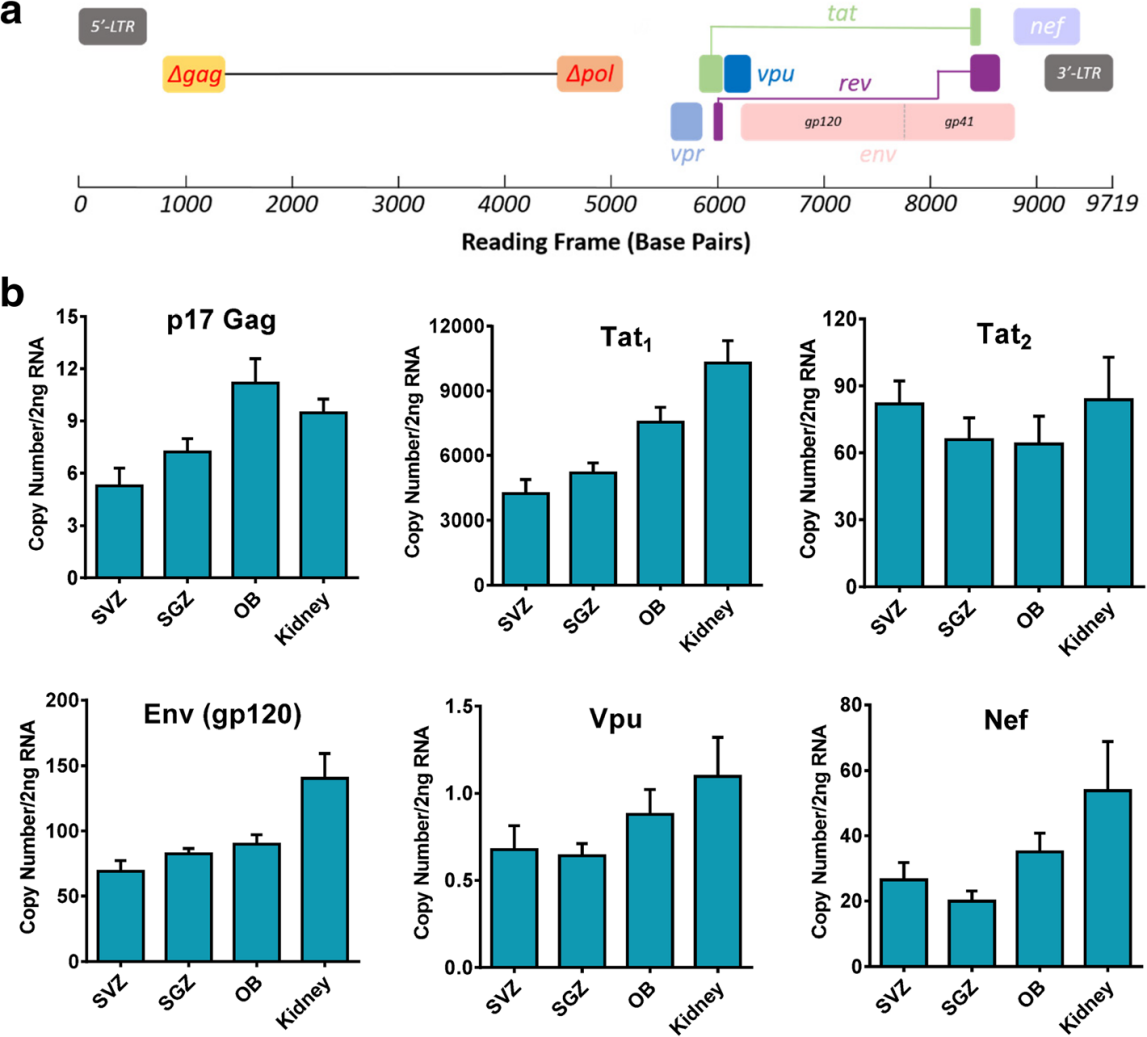
HIV-1 Tg26 transgenic mice express viral mRNA in neurogenic regions. **a** Diagram of the truncated HIV-1 NL4-3 proviral genome in HIV-1 Tg26 mice, showing a deletion of 3.1 kb DNA spanning a majority of the Gag and Pol genes, and the predicted presence of seven viral gene transcripts. **b** RT-qPCR analysis of viral gene transcripts in neurogenic regions of HIV-1 Tg26 mice. Equal amount of RNA from the SVZs, SGZs, olfactory bulbs (OB), and kidneys of HIV-1 Tg26 mice was reverse transcribed, with 2 ng of the generated cDNA used for real-time PCR with primers covering *p17 gag*, *tat*_*1*_ (unspliced variant), *tat*_*2*_ (spliced variant), *env* (*gp120*), *vpu*, and *nef*. Samples were collected from four HIV-1 Tg26 mice (two males and two females), and reactions were run in triplicate. Values are expressed as mean ± SEM

Total RNA was extracted from the brains of four Tg26 mice. Specifically, the SVZs, SGZs, olfactory bulbs, and kidneys were microdissected and stored in Trizol reagent (Thermo Fischer Scientific). The kidneys have been characterized to express proviral transcripts and served as an appropriate positive control for our studies [36, 37]. Littermated WT mice were used as negative controls. The RNA was then purified with the Direct-zol RNA Miniprep Kit according to the manufacturer’s instructions (Zymo Research Cat. # R2052). Equal amounts (100 ng) of RNA from each sample was used for reverse transcription with the High Capacity cDNA Reverse Transcription Kit (Thermo Fischer Scientific Cat.# 4368814), and 2 ng of cDNA was applied for qPCR using specific primers (Table 1, Fig. 1a) targeting partial *gag* (*p17*), *tat* (spliced and unspliced variants), *env* (*gp120*), *vpu*, and *nef* based on previously published reports [43, 44]. Absolute quantification assays were performed using an HIV-1 pNL4-3 plasmid or reverse transcribed 2 or 4 kb HIV-1 cDNA fragments as the standards.

**Table 1:** List of primers used for RT-qPCR and Tg26 mouse genotyping

Gene target	Direction	Sequence (5′ to 3′)
Partial Gag (p17)	T760—forward	GGATAGATGTAAAAGACACCA
	T946—reverse	ACCTGGCTGTTGTTTCCTGTGTC
Env (gp120)	T876—forward	CCGAAGGAATAGAAGAAGAAG
	T691—reverse	AGAGTAAGTCTCTCAAGCGG
Tat_2_ (spliced variant)	T1002—forward	TGGAAGCATCCAGGAAGTCAGCC
	T1003—reverse	TTCTTCTTCTATTCCTTCGGGCC
Tat_1_ (unspliced variant)	T1002—forward	TGGAAGCATCCAGGAAGTCAGCC
	T1007—reverse	GAGAAGCTTGATGAGTCTGACTG
Nef	F3.3—forward	CCGAAGGAATAGAAGAAGAAG
	R3.3—reverse	CTTGTAGCACCATCCAAAGG
Vpu	T1002—forward	TGGAAGCATCCAGGAAGTCAGCC
	R1.3—reverse	GTGGTGGTTGCTTCCTTCC
PCR for mouse genotyping	T361—Tg26 forward	GATCTGTGGATCTACCACACACA
	T363—Tg26 reverse	GCTGCTTATATGCAGCATCTGAG

### Immunohistochemistry (IHC) and immunocytochemistry (ICC)

The following antibodies were used in this study: chicken anti-GFAP (IHC and ICC 1:500, Aves Labs Cat# GFAP, RRID:AB_2313547), Goat Sox2 (IHC and ICC 1:250, Santa Cruz Biotechnology Cat# sc-54517, RRID:AB_2195807), rabbit anti-Ki67 (IHC 1:500, Abcam Cat# ab92353, RRID:AB_2049848), rabbit anti-eGFP (IHC 1:1000, Molecular Probes Cat# A-6455, RRID:AB_221570), goat anti-doublecortin (DCX,1:500, Santa Cruz Biotechnology Cat# sc-8066, RRID:AB_2088494), and rabbit anti-β3-tubulin (Tuj1, ICC 1:1000, Sigma-Aldrich Cat# T2200, RRID:AB_262133).

The IHC and ICC procedures have been conducted in a similar manner as described previously [45]. For ICC, cells were fixed in 4% paraformaldyhyde in phosphate-buffered saline (PBS) for 20 min. After three consecutive PBS washes, the cells were permeabilized for 30 min with 0.5% Triton X-100 mixed with PBS. After permeabilization, the cells were blocked with 2% bovine serum albumin (BSA) dissolved in PBS with 0.1% Triton X-100 for 1 h. NSCs were then treated with primary antibodies diluted in blocking buffer overnight at 4 °C. NSCs were washed three times with PBS and then treated with the appropriate Alexa fluorescent secondary antibodies at room temperature for 1 h, followed by counterstaining with DAPI for 5 min and coverslipping with Fluoroshield (Sigma-Aldrich). Fluorescent confocal microscopy images were acquired and analyzed using the Leica SP8 confocal system.

For IHC, mice were euthanized with an overdose of avertin solution and transcardially perfused with 4% paraformaldehyde. The brains were dissected, postfixed overnight in the same fixative, and cryopreserved with buffered 30% sucrose. A series of coronal sections of brain at 40 μm thickness were cut cryostatically and stored at − 20 °C. Then, standard multiple-labeled fluorescent IHC was performed. Briefly, the floating brain sections (40 μm) were washed three times with 0.5% Triton X-100/tris-buffered saline (TBS) and blocked for 30 min in blocking buffer containing 2% BSA in TBS with 0.5% Triton X-100. Then, primary antibodies were added and the sections were incubated overnight at 4 °C. After three washes, the brain sections were treated with the corresponding Alexa fluorescent labeled secondary antibodies in blocking buffer for 1 h at room temperature. After washing and DAPI counterstaining (Sigma Aldrich Cat. #D9542), brain sections were mounted onto glass microscope slides and then coverslipped with Fluoroshield (Sigma Aldrich Cat. #F6182). Image acquisition and analysis was also performed with the Leica SP8 confocal system.

To assess in vivo hippocampal neurogenic dynamics, specific antibody combinations were used to label different cellular types. Generally, GFAP and Sox2 co-localization is used to histologically mark NSCs, while Sox2 immunoreactivity only marks NPCs [46]. Additional Ki67 immunoreactivity would help distinguish quiescent NSCs from the actively dividing NSCs [47]. DCX is a microtubule-associated protein that is prominently expressed in neuroblasts, and becomes lost once the neuroblast terminally differentiates into a neuron, which usually expresses NeuN and β3-tubulin. All these cell types during adult neurogenesis were quantified in an unbiased stereological manner as described previously [24, 27, 46].

### In vitro NSC stemness and differentiation assay

The isolation and culture of NSCs from 8- to 12-week-old WT or Tg26 mice were performed as described previously [45, 48]. First, the mice were euthanized by cervical dislocation, and their brains were rapidly dissected and placed in dissection wash buffer (Hanks buffered salt solution containing 0.6% glucose, 10 mM HEPES, 2 mM l-glutamine, and 1% penicillin/streptomycin, all from Corning CellGro). Their SVZs were microdissected, placed in wash buffer, and digested with Type IV Collagenase (Worthington Biochemical, Cat. # CLS-4) and 0.05% Trypsin (Thermo Fisher Scientific Cat. # 25300054) diluted in wash buffer. After tissue digestion, the SVZ tissue was gently triturated with a P1000 pipette tip approximately 20 times until a homogenous cell suspension was achieved. The dissociated cells were then seeded in NSC culture media consisting of 0.2% heparin (Stem Cell Technologies Cat. # 07980) (diluted 10,000×), 20 ng/ml epidermal growth factor (EGF) (Stem Cell Technologies Cat.# 78016), 10 ng/ml basic fibroblast growth factor (bFGF) (Stem Cell Technologies Cat.# 78003), 2 mM l-glutamine (Corning), and 1% penicillin/streptomycin (Corning) in DMEM/F12 media (Corning). Primary neurospheres were grown in culture for 7 days, followed by microscopy and imaging to determine size and quantity of the neurospheres present.

The primary neurospheres were further digested with Accutase (Sigma) to obtain a single cell suspension for in vitro lineage differentiation studies as described previously [45, 48]. Briefly, 20,000 cells were seeded in 4 well Matrigel-coated 8-well chamber slides per genotype in NSC proliferation media. The next day, neural differentiation was induced by removal of bFGF and EGF [45, 49] and maintained for 5 days before fixation in 4% paraformaldyhyde. ICC was performed with antibodies against DCX (neuroblasts), β3-tubulin (neurons), and GFAP (astrocytes). Confocal images were taken at five to eight fields per well, and the percentage of differentiated cells over DAPI-positive cells was quantified in a genotype-blinded manner.

### Retrovirus production and stereotaxic injection into the hippocampus

Packaging of the eGFP-containing retrovirus was performed in GP2-293 cells (Clonetech Cat. # 631458) in accordance with previously established protocols [50, 51]. Briefly, 15 μg of the pUX-eGFP vector (a gift from Dr. Shaoyu Ge at State University of New York) was co-transfected via the calcium phosphate method with 15 μg of pVSVG per 100 mm dish when the GP2-293 cells were 70–80% confluent. At 48 and 72 h after transfection, the cell media, which contained infectious retroviral particles, was collected and pooled together for concentration via ultracentrifugation at 25,000 RPM for 2 h at 4 °C. The viral pellet was resuspended in PBS and agitated overnight on a shaker in 4 °C. Viral titering was performed on HEK293T cells, with titers ranging up to 1 × 10^8^ colony-forming units (cfu)/ml.

Stereotaxic surgery was performed on 10-week-old mice. Before the injections, the mice were anesthetized with Avertin (Sigma Aldrich Cat. #T48402-25G) (180 μl/10 g body weight). Surgery was initiated when lack of tactile response was observed. The mice were mounted onto a stereotaxic frame, and the hair on the head was shaved to expose the scalp. After dissection of the scalp, four shallow holes were drilled into the skull using a dental drill (0.5 mm drill bit) at the following stereotaxic coordinates:

- AP, − 2.0 mm from bregma; lateral, ± 1.5 mm
- AP, − 3.0 mm from bregma; lateral, ± 2.5 mm

After the holes were drilled, 2 μl of concentrated retrovirus was injected at each of these stereotaxic coordinates with a 10-μl Hamilton microsyringe:

- AP, − 2.0 mm from bregma; lateral, ± .5 mm; ventral, − 2.0 mm
- AP, − 3.0 mm from bregma; lateral, ± 2.5 mm; ventral, − 3.0 mm

After the stereotaxic injections were completed, the wound was closed with sterile surgical sutures, and the mice were returned back to their cages until further analysis at 4 weeks after injection.

### Confocal imaging and Sholl/dendritic spine analysis of retroviral eGFP-labeled dentate granule neurons

Imaging of eGFP-labeled dentate granule neurons for Sholl analysis and dendritic spine analysis was performed using a Leica SP8 confocal microscopy system. For Sholl analysis, images were taken at a × 63 objective with a Z-stack thickness of 1.0 μm. For dendritic spine analysis, images were acquired with a × 63 objective with a Z-stack thickness of 0.2 μm. For each granule neuron, three to four apical dendritic segments totaling approximately 200 μm were used for dendritic spine analysis as described previously [52]. Neurolucida360 software (MBF Bioscience, RRID:SCR_001775) was used for Sholl and dendritic spine analysis, using maximum projection Z-stack images of the granule neurons or dendritic spines [53].

### Statistical analysis

All statistical analysis was performed using GraphPad Prism 6.0. An unpaired two-tailed Student’s *t* test was performed between two groups of different treatments or genotypes. The *p* value thresholds for statistical significance were set at < 0.05, < 0.01, and < 0.001.

## Results

### HIV-1 Tg26 transgenic mice express viral mRNA in the neurogenic regions

Various transgenic animal models have been used for the study of HIV-1 [3]. While the HIV-1 transgenic rat has shown varying levels of proviral gene expression in the CNS [54], no studies to date have characterized the extent of proviral gene expression in the brains of HIV-1 Tg26 mice. To this end, absolute quantitative RT-PCR was performed using the primers targeting specific viral transcripts (Fig. 1a, Table 1) in the neurogenic regions. With this method, varying copy numbers of transcripts for partial *gag* (p17), *tat* (unspliced and spliced variants), *env* (gp120), *vpu*, and *nef* were detected in Tg26 mice (Fig. 1b), specifically in the SVZ and SGZ neurogenic regions. The unspliced *tat1* transcript showed the highest level of expression in all the neurogenic zones and the kidney. The e*nv* (*gp120*), the spliced *tat2*, and the *nef* transcripts showed the second highest levels of expression, though it is interesting to note that their expression levels were relatively similar. Finally, the *p17 gag* transcript and the *vpu* transcript showed the lowest levels of expression in the neurogenic zones and the kidney. However, *C*_*t*_ values for the *vif* or *vpr* transcripts were not detected in the tested tissues or the kidney samples, most likely due to their relatively low expression levels. The PCR efficiency for *vif* and *vpr* was validated using the cDNA, which was reverse transcribed from the RNA of HIV-1 latent J-Lat cells. Interestingly, the expression levels of all the viral gene transcripts in the kidney were less than twofold higher than the expression levels in the neurogenic zones in the Tg26 mice with a pure C57BL/6J background.

### Neural stem cells (NSCs) from HIV-1 Tg26 transgenic mice exhibit in vitro NSC quiescence and neuronal lineage differentiation deficits

**Fig. 2:**
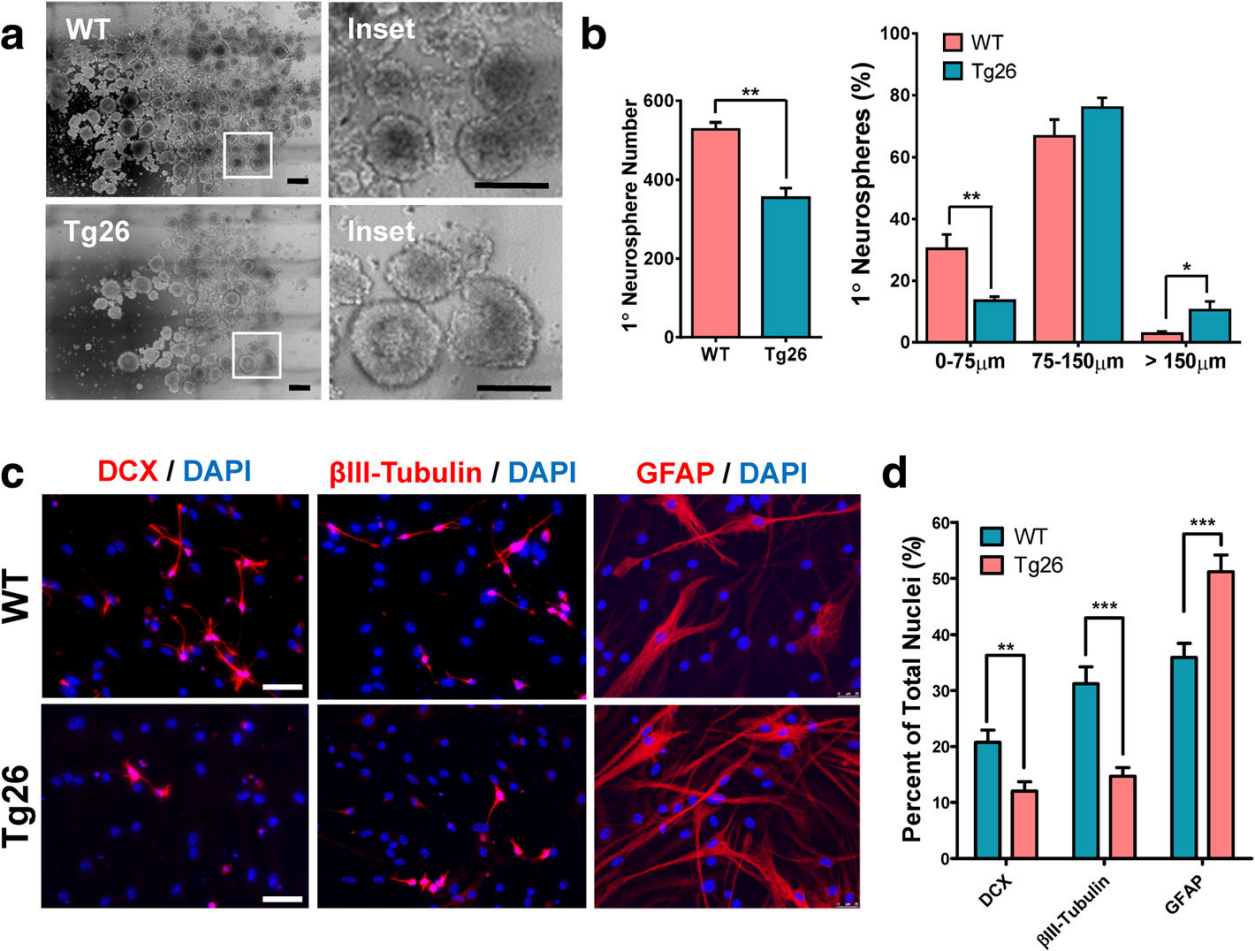
In vitro stemness and neural lineage differentiation are impaired in HIV-1 Tg26 transgenic mice. **a**, **b** Fewer number of primary neurospheres but higher proportion of larger sized neurospheres in Tg26 mice. Representative tiled images of SVZ-derived primary neurospheres at 7 days in vitro are shown (**a**), and the number of the size stratified primary neurospheres was quantified using ImageJ software (**b**). Data is presented as the mean ± SEM from four mice per genotype. **c**, **d** Reduced neuronal lineage differentiation but increased astroglial lineage differentiation in Tg26 mouse NSCs/NPCs. Dissociated NSCs/NPCs from primary neurospheres were differentiated for 5 days followed by immunocytochemistry with cell lineage-specific antibodies to assess for neuroblast (DCX) and neuronal (β3 tubulin) differentiation, and astrocytic (GFAP) formation (**c**). Tg26 mouse NSCs were unable to form as many neuroblasts or neurons as wild-type (WT) mouse NSCs, but instead formed more astrocytes (**d**). The quantitative differentiation data is presented as the Mean ± SEM from 4 mice (2 males and 2 females per genotype), with five to eight random fields per mouse for each cellular marker. Scale bar in **a**, 300 μm; inset, 150 μm. Scale bar in **c** 50 μm. *p < 0.05, **p < 0.01, ***p < 0.001

To explore whether Tg26 transgenic mice exhibit early and late differentiation deficits, primary neurospheres were cultured from both Tg26 mice and WT littermated mice in accordance with previously established protocols [45]. The stemness assay [45] revealed significantly fewer number of primary neurospheres in Tg26 mice than that of littermated WT mice (Fig. 2a, b) (*t*_6_ = 5.836; *p* = 0.0011). When the primary neurospheres were stratified by size (Fig. 2b), Tg26 mouse NSCs formed significantly less smaller sized neurospheres (< 75 μm) (*t*_5_ = 4.049; *p* = 0.0098), but significantly more large-sized neurospheres (> 150 μm) (*t*_6_ = 2.829; *p* = 0.0281). Since larger sized neurospheres typically contain more NSCs and smaller sized neurospheres contain more NPCs [45, 48, 54, 55], these results suggest that Tg26 NSCs have significantly increased quiescence, implying that Tg26 mouse NSCs exhibit deficits in initial differentiation of NSCs to NPCs.

After the initial differentiation of NSCs to NPCs, the differentiation of NPCs to neuroblasts and finally the immature/mature neural cells continues throughout the middle/late stages of adult neurogenesis [16]. To explore whether the chronic stress induced by HIV-1 viral proteins affects neural lineage differentiation, multi-labeled ICC analysis of cultured differentiated NSCs was performed. As shown in Fig. 2c, d, Tg26 NSCs were unable to form as many neuroblasts (*t*_48_ = 3.222; *p* = 0.0023) and neurons (*t*_58_ = 6.4989; *p* < 0.0001) as WT NSCs. However, Tg26 NSCs had an increased incidence of astrocyte formation (*t*_58_ = 3.889; *p* = 0.0003). These results show that Tg26 NSCs exhibit hampered neuronal differentiation and aberrant astrocytic differentiation.

### HIV-1 Tg26 mice exhibit hippocampal neurogenic deficits in vivo

**Fig. 3:**
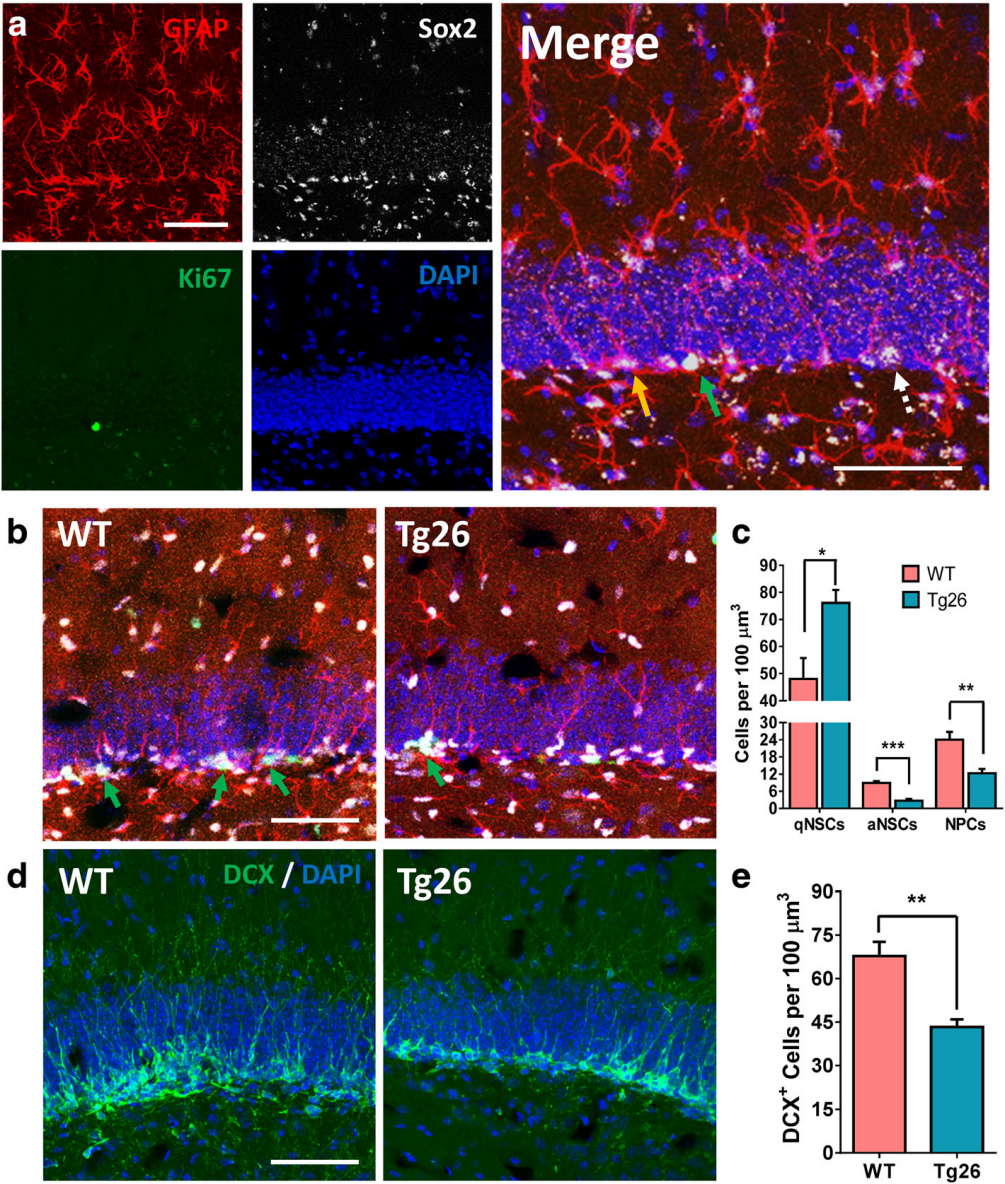
Tg26 mice exhibit hippocampal neurogenesis deficits. **a** Representative confocal image showing quiescent NSCs (qNSCs, orange arrow, GFAP^+^/Sox2^+^/Ki67^−^), active NSCs (aNSCs, green arrow, GFAP^+^/Sox2^+^/Ki67^+^), and NPCs (dashed arrow, GFAP^−^/Sox2^+^) in the SGZ. **b** Comparative SGZ images from WT and littermated Tg26 mice showing less aNSCs (green arrows) in Tg26 mice. **c** Stereological quantification revealed significantly increased numbers of qNSCs, decreased numbers of aNSCs, and decreased numbers of NPCs in Tg26 mouse SGZs. **d** Representative confocal images showing DCX-positive neuroblasts in WT and Tg26 SGZs. **e** Stereological quantification revealing less neuroblast formation in Tg26 mice. Data in **c** and **e** represent the mean ± SEM from five to six mice per genotype (all females). Scale bars in **a**, **b**, and **d**, 50 μm. *p < 0.05 and **p < 0.01 indicate statistical changes compared with the corresponding WT SGZ

To validate the in vitro findings that Tg26 NSCs exhibit stemness and neuronal differentiation impairments, multi-labeled fluorescent IHC with cell-specific markers and confocal imaging analysis (Fig. 3a, b) were performed in serial brain sections from WT and Tg26 mice. Stereological quantification (Fig. 3c) showed that the SGZs of Tg26 mice had significantly higher numbers of quiescent NSCs (*t*_8_ = 2.447; *p* = 0.0401) and significantly lower levels of NSC proliferation (*t*_9_ = 3.523; *p* = 0.0065). Additionally, Tg26 mouse SGZs showed fewer numbers of NPCs than WT mouse SGZs (*t*_7_ = 4.04; *p* = 0.0049). Finally, Tg26 mouse SGZs had lower neuroblast formation (Fig. 3d, e), as depicted by significantly fewer numbers of DCX immunostained cells in the SGZ (*t*_8_ = 3.061; *p* = 0.0156). Altogether, these studies suggest that Tg26 mice have significantly increased NSC quiescence and impaired initial differentiation of NSCs into NPCs as well as decreased neuronal lineage differentiation in vivo. This further confirms the in vitro results from the primary neurosphere assays and the in vitro lineage differentiation assays.

### Newborn dentate granule neurons in HIV-1 Tg26 mice exhibit dendritic arborization deficits, decreased dendritic length, and decreased dendritic spine density

**Fig. 4:**
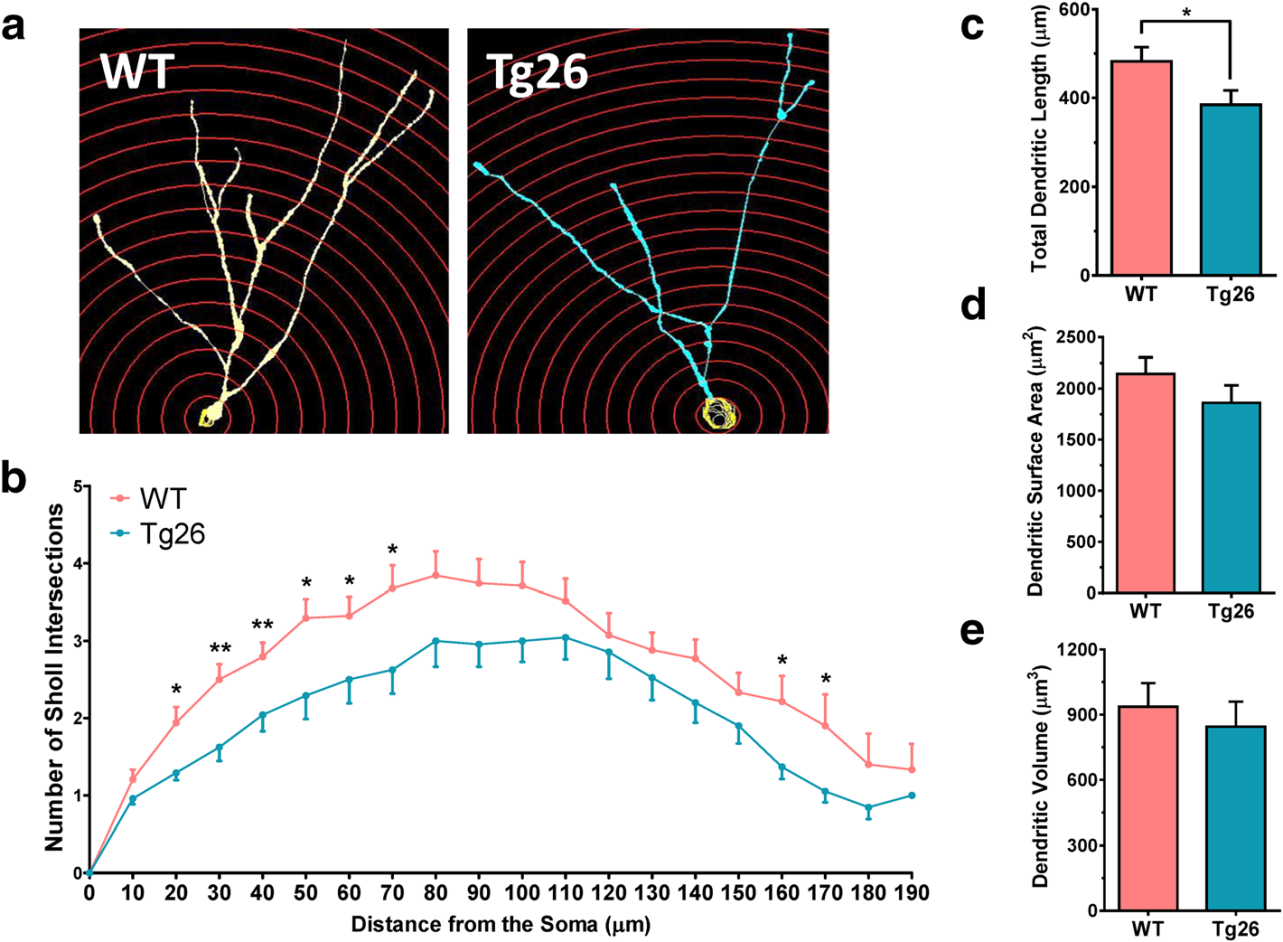
Newborn dentate granule neurons in Tg26 mice have lower dendritic complexity and length. **a** Representative retroviral eGFP-labeled dentate granule neurons from WT and Tg26 mice with the Sholl analysis. Each Sholl circle increases in diameter by 10 μm from the cell soma. **b** Quantitative data of Sholl intersections. **c**–**e** Retroviral eGFP-labeled dentate granule neurons in WT and Tg26 mice were also assessed for total dendritic length (**c**), dendritic surface area (**d**), and dendritic volume (**e**). Data is presented as the mean ± SEM from four mice (three males and one female per genotype), with six to eight granule neurons being analyzed per mouse. *p < 0.05 and **p < 0.01 indicate statistical changes compared with corresponding WT granule neuron

**Fig. 5:**
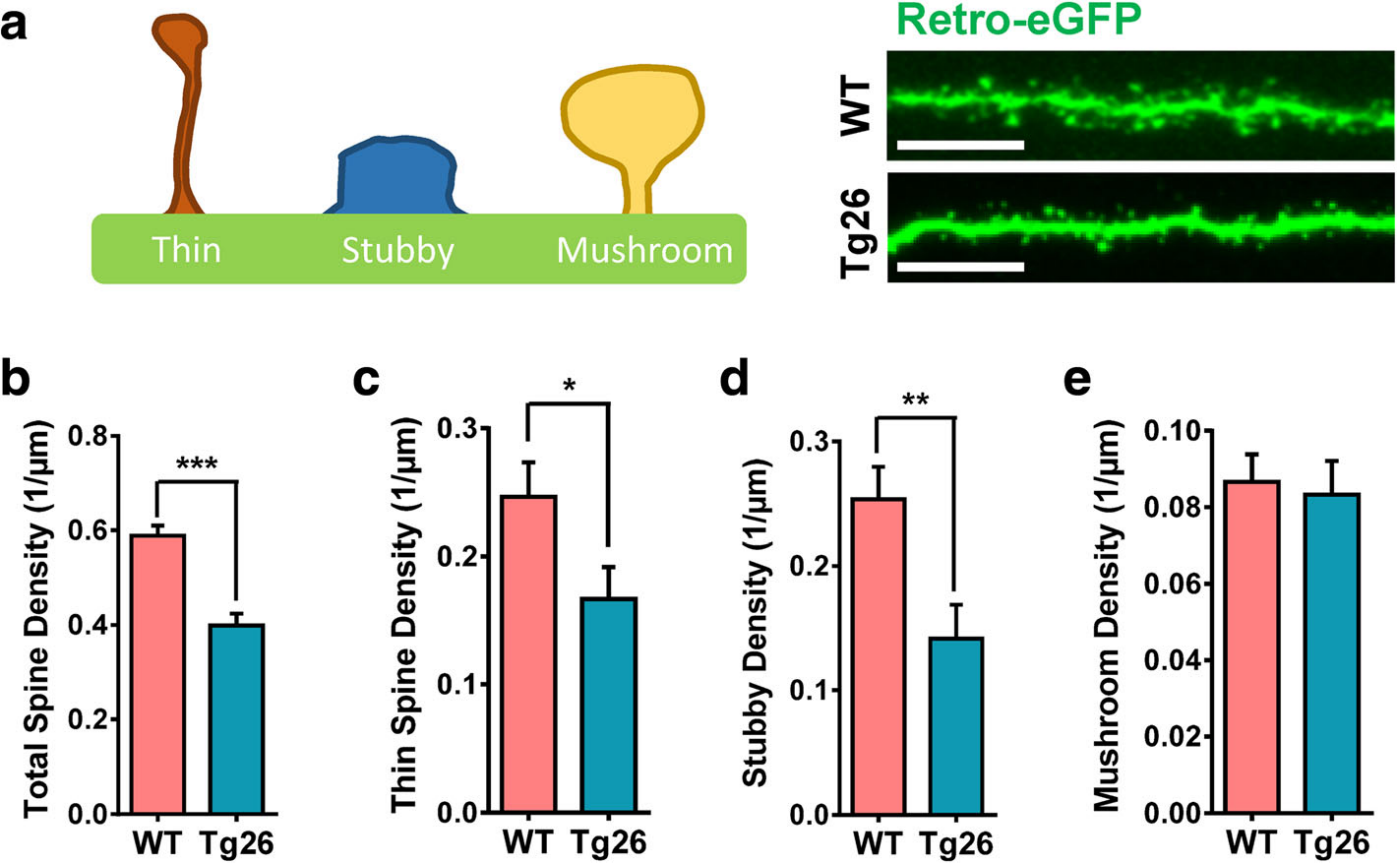
Newborn dentate granule neurons in Tg26 mice harbor decreased apical dendritic spine density. **a** Diagram of dendritic spine types and representative images of retroviral eGFP-labeled apical dendritic arbors from WT and Tg26 granule neurons. **b**–**e** Quantitative analysis of total dendritic density as well as densities for specific spine types. Data is presented as the mean ± SEM from four mice (three males and one female per genotype), with at least 200 μm of apical dendritic arbors from six to eight granule neurons being analyzed per mouse. Scale bar in **a**, 6.25 μm. *p < 0.05, **p < 0.01, and ***p < 0.001 indicate statistical changes compared with the corresponding WT dendritic arbor

Both the in vitro and in vivo studies, as described above, have shown that HIV-1 transgenic viral proteins significantly perturbed NSC initial differentiation and neuroblast/neuronal linage differentiation during adult neurogenesis. Next, experiments were performed to determine whether chronic stress from HIV-1 viral proteins in Tg26 mice had effects on late-stage neuronal maturation. Therefore, the maturation of newborn granule neurons in the dentate gyrus was analyzed using a well-established retroviral eGFP labeling technology [24, 50, 51]. Here, actively dividing cells (i.e., active NSCs, proliferating NPCs, and a few dividing neuroblasts) were instantly labeled with the eGFP reporter after stereotaxic injection of the retrovirus into the dentate gyrus. The fate of the labeled cells was mapped at different times after injection as described previously [24, 50, 51]. Studies have shown that maturation of newborn granule neurons from SGZ NSCs can be viewed by eGFP IHC and confocal image analysis approximately 4 weeks after retroviral injection in mice [50]. To identify any potential effects of chronic HIV-1 viral protein stress on dendritic arborization and spinogenesis during neuronal maturation [50], Sholl analysis on eGFP-labeled granule neurons in SGZ of WT and Tg26 mice at 4 weeks after retroviral injection into the dentate gyrus was performed using Neurolucida360 software (Fig. 4a). The eGFP-labeled newborn neurons in Tg26 mice exhibited significantly lower levels of dendritic arborization and complexity (Fig. 4b) at 20 μm (*t*_56_ = 2.557; *p* = 0.0133), 30 μm (*t*_56_ = 3.116; *p* = 0.0029), 40 μm (*t*_64_ = 2.753; *p* = 0.0077), 50 μm (*t*_56_ = 2.590; *p* = 0.0122), 60 μm (*t*_56_ = 2.115; *p* = 0.0389), 70 μm (*t*_56_ = 2.401; *p* = 0.0197), 160 μm (*t*_31_ = 2.492; *p* = 0.0182), and 170 μm (*t*_27_ = 2.411; *p* = 0.0230) away from the cell soma. This decrease in dendritic complexity also correlated with Tg26 granule neurons having a lower dendritic length (Fig. 4c) (*t*_48_ = 2.035; *p* = 0.0474). However, total dendritic surface area and dendritic volume remained unchanged (Fig. 4d, e). In addition to dendritic arborization deficits, several studies have shown that HIV-1-infected patients exhibit synaptodendritic damage, even while on ART [56, 57]. To examine whether Tg26 mouse dentate granule neurons have any changes in dendritic spine integrity, dendritic spine density analysis was conducted to assess any differences in total spine density, thin spine density, stubby spine density, and mushroom spine density (Fig. 5a). The eGFP-labeled granule neurons from HIV-1 Tg26 mice had significantly less apical dendritic spine densities (*t*_43_ = 5.681; *p* < 0.0001) compared to that from WT mice (Fig. 5b). Dendritic spines come in a variety of shapes and sizes, with the more common classifications being designated as thin spines, stubby spines, and mushroom-shaped spines [53]. Quantification of these specific spine types revealed that Tg26 granule neurons had a significantly lower thin spine density (Fig. 5c) (*t*_40_ = 2.047; *p* = 0.0473) and stubby spine density (Fig. 5d) (*t*_41_ = 2.884; *p* = 0.0062). However, quantification of mushroom spine density revealed that there were no differences between WT and Tg26 granule neurons (Fig. 5e). This data suggests that low-level chronic HIV-1 expression induces synaptodendritic damage in a manner similarly observed in HIV-1-infected patients [5].

## Discussion

In the ART era, HIV-1 infection continues to elicit mild to moderate forms of HAND, more notably ANI and MND [5, 6]. Many mechanisms have been characterized that explain some of the pathogenesis underlying ANI/MND, but recently, compromised adult neurogenic changes have been proposed as a newer mechanism for HIV-induced CNS injury [2, 3, 27, 31]. The salient findings of this study include (1) varying levels of HIV-1 proviral transcripts in the neurogenic zones of HIV-1 Tg26 mice, (2) deficits in initial NSC differentiation into NPCs in vitro and in vivo, (3) decreased neuronal lineage differentiation in vitro and in vivo, and (4) impairment of neuronal maturation and spinogenesis in newborn dentate granule neurons. These findings solidify the HIV-1 Tg26 mouse model as an appropriate approach to study the milder forms of HAND, which are clinically relevant to the HIV-1 latently infected population under ART today [6, 58].

Several pioneering studies have shown mosaic levels of HIV-1 proviral expression in peripheral organs such as the spleen, thymus, kidney, and muscle of Tg26 mice [36, 37, 59]. However, no previous studies have assessed viral gene expression levels in Tg26 mouse brains. To our knowledge, we are the first group to show the presence of the unspliced and spliced transcripts of HIV-1 viral genes not just in the brain, but specifically in different neurogenic niches. Our RT-PCR analysis revealed mosaic levels of p17 Gag, Tat (unspliced and spliced variants), Env (gp120), Vpu, and Nef transcripts in the different neurogenic zones. These mosaic levels of proviral gene transcript expression are consistent with previous reports in HIV-1 transgenic rats using the same truncated pNL4-3 construct with all the transcripts driven by the HIV-1 5′-LTR promoter [54]. Interestingly, the unspliced Tat1 transcript showed a dramatically higher expression level than the spliced Tat2 transcript and other viral transcripts, implying that Tat1 might be the major contributor to CNS neurotoxicity [60–62]. Due to replication deficiency, the expression of these viral transcripts is relatively lower but constitutively persistent, implicating its clinical relevance to chronic HIV-1 infection in the ART era. The presence of HIV-1 viral transcripts in neurogenic zones also supports an earlier clinical finding by in situ hybridization in the SVZ and SGZ of a post-mortem patient with severe NeuroAIDS, showing that HIV-1 RNA transcripts localized to the neurogenic zones [18]. Further analysis using more sensitive technologies such as RNA/DNAscope [63, 64] and barcoded single-cell sequencing [65, 66] are warranted to distinguish the cellular distribution of HIV-1 viral transcripts in the neurogenic zones.

Most cases of HAND consist of subtle or milder forms of neurocognitive deficits. Since neurogenic defects have been shown to contribute to developing many subtle neurocognitive changes after CNS injury or neurodegenerative diseases [67, 68], it has been hypothesized that neurogenic impairment may contribute to the more subtle forms of HAND. This neurogenic impairment by HIV-1 single viral proteins has been demonstrated by several previous studies using in vitro cell cultures [25, 28, 31] and in vivo transgenic animal models [22, 24, 27, 56, 69]. For example, treating cultured NSCs/NPCs with Tat has been shown to inhibit NSC proliferation and differentiation through attenuation of the ERK pathway [70] and the p38-MAPK pathway [71] or through activation of the Notch pathway [27]. Additionally, treating NSCs/NPCs with gp120 suppresses NSC proliferation via activation of p38 MAPK pathway [25]. In this study, we performed a more comprehensive evaluation of impaired neurogenesis at both the in vitro and in vivo levels using a clinically relevant HIV-1 transgenic mouse model that harbors seven of nine HIV-1 viral genes. Our in vitro NSC stemness assays revealed a significantly diminished NSC pool in Tg26 mice compared to their WT littermates, as assessed by the reduced number of the primary neurospheres generated. Additionally, we found that chronic HIV-1 viral protein stress increased the formation of larger sized primary neurospheres, implying the impairment of initial differentiation of NSCs into NPCs. Primary neurospheres contain a mixed population of de novo NSCs and NPCs [45, 48], and larger sized neurospheres represent the existence of the tri-potential, self-renewing NSCs [55, 72]. These in vitro findings were validated in vivo, as Tg26 mouse SGZs had a higher level of quiescent NSCs, while having a diminished proliferating NSC population as well as a lower NPC pool. This increased NSC quiescence due to the hampered initial activation and differentiation into NPCs is consistent with previous reports on cultured NSCs treated with Tat and other viral proteins [70, 71, 73–75] and using in vivo animal models [20, 25, 73, 74, 76].

NSCs/NPCs terminally differentiate into neurons and glial cells. This lineage differentiation process is regulated by a series of environmental niche factors. Inflammatory mediators such as cytokines and chemokines have been widely shown to regulate this tri-potential lineage differentiation pattern in varying ways. The direct effects of chronic HIV-1 infection or viral proteins on NSC lineage differentiation have yet to be comprehensively understood. Our in vitro and in vivo neural lineage differentiation studies have shown that Tg26 NSCs have a decreased affinity towards neuronal lineage differentiation, but exhibit increased astroglial lineage differentiation. Similar effects have been observed in cultured NSCs/NPCs treated with Tat [70, 77] or direct HIV-1 infection [76], as well as in mice with HIV-1 encephalitis [78], doxycycline-inducible GFAP-Tat transgenic mice [27], and GFAP-gp120 mice [24, 25, 79]. However, a recent in vitro study has shown that active HIV-1 infection of human NSCs promotes both neuronal and astroglial lineage differentiation, when compared to uninfected human NSCs [31]. One possible explanation could be intrinsic mechanistic difference between various HIV-1 strains: the CCR5-tropic HIV-1 BaL viral strain in previous study [31] vs the CXCR4-tropic NL4-3 strain in Tg26 mice. CCR5-tropic viruses represent the predominant viral population right after initial infection, but CXCR4 tropic viruses are associated with progressive CNS injury [80, 81]. In the clinical setting, CXCR4 tropic viruses may contribute more to the milder forms of HAND than CCR5 tropic viruses [3], which would make our in vivo HIV-1 Tg26 mouse model more clinically relevant. Another explanation may be the type of infection being performed: active infection vs. low-level chronic/latent infection. The supernatant from HIV-infected peripheral blood mononuclear cells may induce a more robust and confounding effect on NSC differentiation due to the combination of active virial particles, HIV-1 viral proteins, growth factors, and inflammatory mediators secreted by the infected cells [31].

Adult neurogenesis is a process that describes the generation of neural lineage cells from NSCs and NPCs. However, it is important to recognize the neuronal maturation and synaptic integration into this process. As of now, no studies have examined the effects of chronic HIV-1 infection and proviral protein stress on newborn neuronal formation and maturation. Retroviral eGFP labeling of newborn granule neurons in the dentate gyrus revealed that Tg26 mouse granule neurons have significantly decreased dendritic morphology, decreased total dendritic length, and decreased dendritic spine densities. These findings are notable, as these are the first to implicate the combined HIV-1 viral proteins in dendritic arborization deficits in newborn dentate granule neurons. Our observed decrease in dendritic spine density in dentate granule neurons is consistent with a previous study analyzing dendritic spine damage in layer II/III pyramidal prefrontal cortical neurons of both HIV-1 transgenic rats and gp120 infused rats [52], as well as aberrant dendritic development of newborn neurons in GFAP-gp120 transgenic mice [24]. Our Neurolucida360 studies further elaborated on the detrimental effects on various types of dendritic spines (thin, stubby, or mushroom spines) instead of only total dendritic spine density [52]. Additionally, our Sholl analysis identified dendritic morphological deficits of newborn hippocampal dentate granule neurons in Tg26 mice, while no differences in pyramidal cortical neuron dendritic length or branching were observed in HIV-1 transgenic rats [52]. This abnormal dendritic arborization in HIV-1 Tg26 mouse dentate granule neurons also supports a previous study using similar retroviral labeling technology of newborn dentate granule neurons in GFAP-gp120 mice [24]. However, further electrophysiological and circuit tracing experiments are needed to assess any functional differences in the newborn matured granule neurons between WT and Tg26 mice. Additionally, the molecular mechanisms behind the abnormal dendritic spine density in HIV-1 Tg26 mice remain to be determined.

## Conclusions

Our in vitro and in vivo studies demonstrate that HIV-1 Tg26 transgenic mice manifest early-state and late-stage neurogenic deficits, when compared to their WT littermates. Additionally, these neurogenic deficits lead to deficient dentate granule neuron morphology as well as decreased dendritic spine densities. These neurogenic deficits may possibly play a role modulating learning and memory function in these mice, which is an interesting topic for further investigation.

## Abbreviations

ANI: Asymptomatic neurocognitive impairment
ART: Antiretroviral treatment
*bFGF*: Basic fibroblast growth factor
CNS: Central nervous system
DCX: Doublecortin
EGF: Epidermal growth factor
eGFP: Enhanced green fluorescent protein
GFAP: Glial fibrillary acidic protein
HAD: HIV-associated dementia
HAND: HIV-associated neurocognitive disorders
HIV: Human immunodeficiency virus
ICC: Immunocytochemistry
IHC: Immunohistochemistry
LTR: Long terminal repeat
MND: Mild neurocognitive disorder
NPCs: Neural progenitor cells
NSCs: Neural stem cells
PBS: Phosphate-buffered saline
RT-PCR: Reverse transcription-polymerase chain reaction
SGZ: Subgranular zone
SVZ: Subventricular zone
TBS: Tris-buffered saline
TG: Transgenic
WT: Wild-type
